# Microbial nitrification, denitrification and respiration in the leached cinnamon soil of the upper basin of Miyun Reservoir

**DOI:** 10.1038/srep42032

**Published:** 2017-02-06

**Authors:** Wen Xu, Yan-Peng Cai, Zhi-Feng Yang, Xin-An Yin, Qian Tan

**Affiliations:** 1 State Key Laboratory of Water Environment Simulation, School of Environment, Beijing Normal University, Beijing, 100875, China; 2 Beijing Engineering Research Center for Watershed Environmental Restoration & Integrated Ecological Regulation, School of Environment, Beijing Normal University, Beijing, 100875, China; 3 Institute for Energy, Environment, and Sustainable Communities, University of Regina, Research Drive, Beijing 100083, China; 4 School of Water Resources & Civil Engineering, China Agriculture University, Beijing 100083, China

## Abstract

Leached cinnamon soil is the main agricultural soil distributed in the North China Plain. In this research, leached cinnamon soil samples were collected in the upper basin of Miyun Reservoir (northeast of Beijing, China). The BaPS method (Barometric Process Separation) was applied to measure nitrification, denitrification and respiration rates. The rates of nitrification, denitrification and respiration were 0–120.35 μg N/kg SDW h, 0–246.86 μg N/kg SDW h and 0.17–225.85 μg C/kg SDW h (Soil Dry Weight, SDW), respectively. The emission rates of CO_2_ and N_x_O_y_ through nitrification, denitrification and respiration were 1.00–547.80 and 6.00–4850.65 μmol/h, respectively. The analysis of relationships between nitrification, denitrification and respiration rates indicated that these three microbial processes were interacted, which posed impacts on soil nitrogen availability. As indicated by the results, C:N ratio coupled with 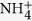 content could be taken as the indicators of 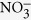 content, which is usually the predominant form of N available to plants growing in soil. Results showed that 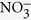 content was the highest (i.e., >62.4 mg/kg) when C:N ratio was 5.30–8.40, meanwhile 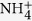 content was 3.71–4.39 mg/kg. Nevertheless, 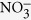 content was the lowest (i.e., <6.40 mg/kg) when C:N ratio was 9.2–12.10, meanwhile 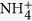 content was 3.41–4.35 mg/kg.

Nitrification, denitrification and respiration are major processes affecting soil greenhouse gases (i.e., CO_2_ and N_2_O) emissions and soil nitrogen availability^1^,^2^,^3^,^4^,^5^,^6^. It has been proven that CO_2_ emission from microbial respiration in soils was in excess of any other terrestrial-atmospheric carbon transactions, which is thus considered as the primary natural source for CO_2_ emission^7^,^8^. The increase of soil CO_2_ emission had the potential of promoting atmospheric CO_2_ levels, which in turn would aggravate global warming^9^,^10^,^11^,^12^,^13^. Recently, a series of studies have addressed microbial respiration under multiple tillage methods, hydraulic properties and vegetation covers^8^,^14^,^15^. A few researchers also found that microbial respiration was strongly influenced by soil nitrogen availability through multiple complex approaches, with both inhibition and promotion effects being observed^16^,^17^,^18^,^19^. Compared to CO_2_, nitrous oxide (N_2_O) was identified to be much more destructive to environment. It is well known that N_2_O has 265 times greater global warming potential than that of CO_2_, and may lead to ozone depletion in the stratosphere^20^. Researchers revealed that agricultural soil was also the major source of N_2_O emission, and almost all the processes that involving N_2_O production were biotic^21^,^22^. From a theoretical point of view, microbial nitrification and denitrification in soils, especially the simultaneous nitrification-denitrification processes under low oxygen conditions, were widely acknowledged to be the most crucial mechanism that was related to a significant amount of N_2_O emission^22^,^23^,^24^. Considerable studies have been launched to determine the factors that are influencing soil N_2_O emission during nitrification and denitrification processes, including soil temperature, moisture, carbon source, COD/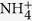 ratio and nitrite accumulation^25^,^26^,^27^. Therefore, identification, analysis and evaluation of the relevant processes such as microbial nitrification, denitrification and respiration in soils are desired not only for mitigating greenhouse gas (GHG) emissions, but also for reducing losses of nutrients in soils and retain effective utilization of fertilizers in agricultural sector^2^,^3^.

Until recently, a number of methods have been proposed to determine the rates of microbial respiration, including alkali absorption^28^,^29^, static chamber-GC and the dynamic chamber-IRGA method^30^. A generally accepted standard method to measure the rates of nitrification and denitrification is the acetylene inhibition technique^31^,^32^. A few researchers also adopted the soil incubation method^33^,^34^ and ^15^N isotope dilution technique^35^ to quantify nitrification and denitrification processes. Nevertheless, the traditional measurement methods regarded nitrification, denitrification and respiration as three independent processes, whereas these three microbial processes have been proven to be interrelated to a certain extent^4^,^17^,^18^. Comparatively, the method of BaPS (Barometric Process Separation) could quantify the rates of nitrification, denitrification and respiration simultaneously through measuring the changes of gas pressure^36^. Thus, the BaPS method has become widely used to determine microbial nitrification, denitrification and respiration rates. Chen *et al*. applied the BaPS method to analyze the variation characteristics of nitrification, denitrification and respiration rates at multiple soil depths, the results indicated that both nitrification and respiration rates decreased with soil depths, but denitrification rates changed negligibly^37^. Shi *et al*. analyzed the impacts of land-use patterns on nitrification and denitrification rates through the BaPS method, the results indicated that land-use patterns could affect microbial activities through altering soil moisture and porosity^38^. Yang *et al*. monitored the nitrification, denitrification and respiration rates of black soil covered by maize under multiple fertilization conditions with the BaPS method. The results manifested that nitrification, denitrification and respiration rates could be accelerated by the increase of fertilizer levels^39^. All of these studies have proved that the BaPS method could help comprehensively analyze soil carbon and nitrogen transformation processes.

Leached cinnamon soil is one of the major cultivated soils distributed in the North China Plain, which is one of the core farm belts in China. Thus studying biogeochemical processes (i.e., nitrification, denitrification and respiration) in leached cinnamon soil is significant for understanding soil carbon and nitrogen transformation. The primary objectives of this paper are to (a) determine the rates of nitrification, denitrification and respiration simultaneously in leached cinnamon soil with the BaPS method, (b) characterize the variations of microbial nitrification, denitrification and respiration rates with varying soil depths and vegetation cover types, (c) analyze the variations of soil/air temperatures with the emissions of CO_2_ and N_x_O_y_ through nitrification, denitrification and respiration processes, and (d) identify the impacts of microbial activities on soil nitrogen availability. The results will be beneficial to understand the transformation processes of carbon and nitrogen in leached cinnamon soil, and will have great implications for controlling greenhouse gases emission from leached cinnamon soil, as well as managing farming practices at a watershed scale.

## Results

### Spatial variations of nitrification, denitrification and respiration rates

**Figure 1:**
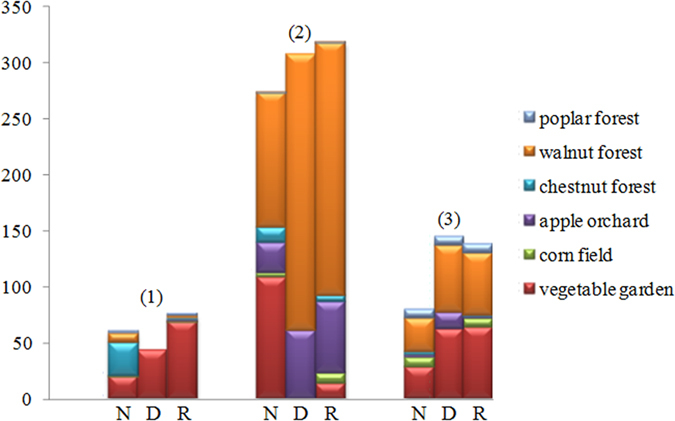
Microbial nitrification, denitrification and respiration rates under various vegetation covers at different soil depth. The unit of nitrification and denitrification is μg N/kg SDW h, and that of respiration is μg C/kg SDW h. N, D and R are short for nitrification, denitrification and respiration, respectively. (1) The first-layer soil: soil sampling depth is 0–10 cm in the vegetable garden and corn field, and is 0–20 cm in the rest vegetation covers; (2) The second-layer soil: soil sampling depth is 10–20 cm in the vegetable garden and corn field, and is 20–40 cm in the rest vegetation covers; and (3) The third- layer soil: soil sampling depth is 20–30 cm in the vegetable garden and corn field, and is 40–60 cm in the rest vegetation covers.

On the whole, microbial nitrification and respiration were detectable in almost all the leached cinnamon soil samples. Results showed that the nitrification rate was 0 to 120.35 μg N/kg SDW h and the respiration rate was 0.17 to 225.85 μg C/kg SDW h (Soil Dry Weight, SDW). However, denitrification was detectable in a small number of soil samples, with potential rates of 0 to 246.86 μg N/kg SDW h. Moreover, denitrification rate was higher than nitrification rate when nitrification and denitrification were co-existence. The rates of microbial nitrification, denitrification and respiration varied considerably with soil depth and vegetation types (Fig. 1).

**Figure 2:**
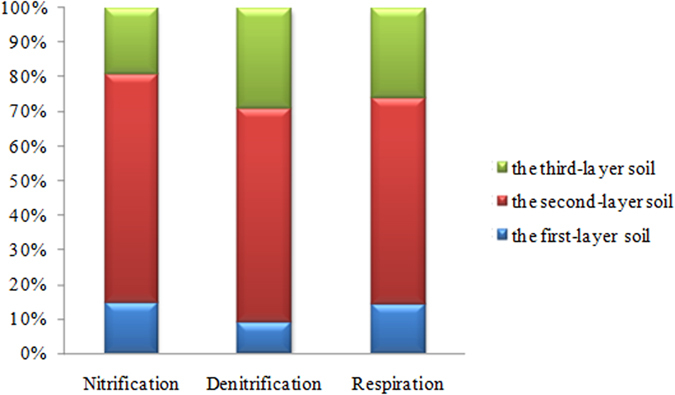
Comparison of microbial nitrification, denitrification and respiration potentials among the three soil layers.

In the first-layer soil, denitrification was detected to be rarely happened, nitrification and respiration were detected to proceed at low rates (i.e., 0 to 29.24 μg N/kg SDW h and 0.98 to 68.95 μg C/kg SDW h, respectively). However, higher nitrification, denitrification and respiration rates (i.e., 120.35 μg N/kg SDW h, 246.86 μg N/kg SDW h and 225.85 μg C/kg SDW h, respectively) were found within the second-layer soil. In the third-layer soil, the rates of nitrification, denitrification and respiration were 2.33 to 30.08 μg N/kg SDW h, 0 to 62.33 μg N/kg SDW h and 1.36 to 64.34 μg C/kg SDW h, respectively. The results showed that nitrification, denitrification and respiration were inhomogeneously distributed among the soil samples within the three soil layers. Specifically, nitrification rates of the second-layer soil samples contributed about 65% to the summation of nitrification rates in all the three soil layers. This tendency was similar with the denitrification and respiration rates in the second-layer soil, which accounted for about 62% and 60% of the summation of denitrification and respiration rates in all the three soil layers, respectively (Fig. 2). It indicated that the second-layer soil was the most suitable place for the happening of microbial nitrification, denitrification and respiration.

With respect to the vegetation types, higher nitrification, denitrification and respiration rates (i.e., 8.46 to 120.35 μg N/kg SDW h, 0 to 246.86 μg N/kg SDW h and 2.1 to 225.85 μg C/kg SDW h, respectively) were found to occur in the vegetable garden and the walnut forest. However, lower nitrification, denitrification and respiration rates (i.e., 0 to 29.24 μg N/kg SDW h, 0 to 61.34 μg N/kg SDW h and 0.17 to 63.28 μg C/kg SDW h) were found to occur in the other four kind of vegetation types (i.e., corn field, apple orchard, chestnut forest and poplar forest).

### The emissions of CO_2_ and N_x_O_y_ through microbial activities

Almost in all the soil samples, we observed obvious variations of gas volume changes (i.e., ΔCO_2_, ΔO_2_, ΔN_x_O_y_ and Δn), which were measured with the BaPS (Barometric Process Separation) method (Table 1). The BaPS method is based on the measuring of CO_2_, O_2_ and total gas balance in a sealed chamber. In such an isothermal and gas tight system, microbial respiration, nitrification and denitrification are the main biological processes responsible for gas volume/pressure changes in the sealed chamber.

Specifically, all the ΔCO_2_ had positive values while all the ΔO_2_ had negative values. ΔCO_2_ represented the total variation of CO_2_ gas volume, which equaled to the production of CO_2_ through denitrification and respiration processes with the amount of CO_2_ assimilated during nitrification being excluded. Therefore, the positive ΔCO_2_ values meant that CO_2_ emission rate was greater than CO_2_ fixation rate, thus the gas volume of CO_2_ in the sealed chamber was increased, which indicated that the leached cinnamon soil samples could be taken as carbon sources. The reason that all the ΔO_2_ had negative values was because nitrification and respiration were oxygen-consumed processes, by which the gas volume of O_2_ in the sealed chamber was reduced. Moreover, it made sense that the Δn and ΔN_x_O_y_ had both positive and negative values. In most of the soil samples, merely nitrification and respiration were detected (Fig. 1), leading to the decrease of total gas volume (negative Δn values). The ΔN_x_O_y_ values were calculated based on the equation (4), therefore, the negative Δn values were likely to bring about negative ΔN_x_O_y_ values. Additionally, in some of the sampling sites, nitrification, denitrification and respiration were simultaneously detected, meanwhile denitrification rate was higher than nitrification and respiration rates (Fig. 1), thus the total gas volume was inflated under this circumstances (positive Δn values), and the ΔN_x_O_y_ values were positive correspondingly. In aggregate, for Δn, ΔCO_2_ and ΔO_2_, positive values stood for an increasing of gas volume and vice versa. However, in terms of the ΔN_x_O_y_ values, the arithmetic signs represented nothing but a result of mathematical operation, meanwhile the numerical value was a reflection of N_x_O_y_ emission rate.

The emission rates of CO_2_ and N_x_O_y_ of the leached cinnamon soil samples were 1.00 to 547.80 μmol/h and 6.00 to 4850.65 μmol/h, respectively. Comparing the results in Fig. 1 and Table 1, we found that higher nitrification, denitrification and respiration rates went with higher emission rates of CO_2_ and N_x_O_y_ in most cases. We also found that once denitrification occurred, the emission rates of CO_2_ and N_x_O_y_ were correspondingly enhanced, which could be explained by the equation (2). Nevertheless, under certain circumstances, the emission rates of CO_2_ or N_x_O_y_ did not correspond to the high nitrification, denitrification and respiration rates.

**Figure 3:**
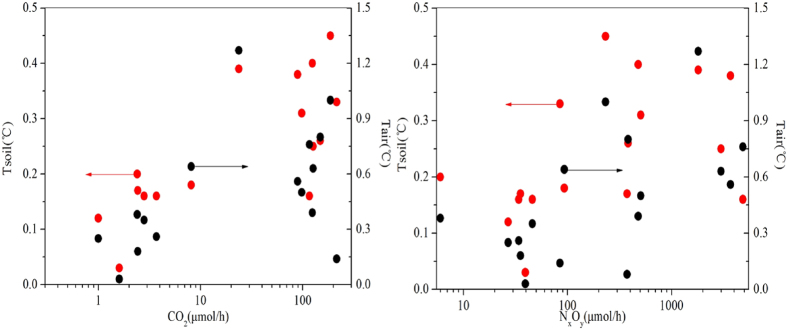
Responses of soil and air temperatures in the sealed chamber to the variation of CO_2_ and N_x_O_y_ emission rates.

It is well known that CO_2_ and N_x_O_y_ were important greenhouse gases causing atmosphere temperature to raise. Therefore, we analyzed the relationship between greenhouse gases (i.e., CO_2_ and N_x_O_y_) emission rates and soil/air temperature variations in the sealed chamber. During the experiment, the sealed chamber was under water bath at a constant temperature of 25 °C, and the laboratory temperature was controlled under 25 °C. Therefore, the increasing of soil and air temperatures (positive ΔT_soil_ and ΔT_air_ values shown in Table 1) in the sealed chamber were the consequences of CO_2_ and N_x_O_y_ emissions from the leached cinnamon soil samples. The increasing of soil and air temperatures in the sealed chamber varied from 0.03 to 0.45 °C and 0.03 to 1.27 °C, respectively. Obviously, a correlation was identified between the elevation of soil and air temperatures in the sealed chamber and the increase of CO_2_ emission rate. By contrasting, the correlation between the N_x_O_y_ emission rate and soil and air temperatures in the sealed chamber was not that obvious (Fig. 3).

### Physical and chemical characters of the soil samples

Soil physical and chemical characters such as pH values, soil water contents, soil organic matter contents, C/N ratios, 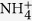 and 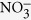 contents of all the soil samples were measured in laboratory, and the results can be found as Supplementary Table S1. Generally, soil samples from the chestnut forest had relatively low pH values ranging from 5.01–6.97, meanwhile soil samples from the vegetable garden had relatively high pH values around 8.30. Soil water contents of the all the soil samples ranged from 7.14% to 17.84%, with an average value of 12.67%. Soil organic matter contents were unevenly distributed, the highest soil organic matter content (i.e., 16.92 g/kg) was about three times larger than the lowest soil organic matter contents (i.e., 5.15 g/kg). The C/N ratios of all the soil samples were between 5.44 and 11.23. All the soil samples had relatively low 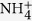 contents, which were 3.06–4.15 mg/kg. However, soil samples from natural forest (i.e., poplar forest) had much lower 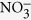 contents (i.e., 8.24–9.37 mg/kg) than those from agricultural lands (i.e., vegetable garden, corn field, apple orchard, chestnut forest and walnut forest).

## Discussions

Microbial respiration is the process that organic matter being decomposed into CO_2_ under aerobic conditions, meanwhile the rate of CO_2_ production provides a direct measure of microbial activity^21^,^40^. However, microbial respiration rate in the first-layer soil mainly kept at a low level (0.98 to 2.14 μg C/kg SDW h), which suggested that microorganism was not active in the first-layer soil. Owing to the low activity of microorganism, nitrification in the first-layer soil was restricted correspondingly, with a rate of 0.84 to 29.24 μg N/kg SDW h. In this research, the depth of the first-layer soil (0 to 10 cm in vegetable garden and corn field, and 0 to 20 cm in the rest vegetation covers) was shallow. Therefore, it was more susceptible to be affected by human activities (i.e., scarification, compaction, and cultivation) that would alter the soil structure. Additionally, in order to avoid nutrient competition, the weeds on the soil surface were almost eradicated up by the farmers, which increased the exposure of soil surface to the atmosphere. Thus accelerating the evaporation of soil moisture as well as attenuating the water retention capacity of fine roots in the first-layer soil. Hence, soil structure and soil moisture were likely to be the main factors that confined the rates of nitrification and respiration. Furthermore, strict soil aeration condition needs to be maintained for the process of denitrification, which can hardly be observed for the first-layer soil. Thus, denitrification was scarcely observed in the first-layer soil.

In our study, the second-layer soil was 10 to 20 cm deep for herbaceous plant and was 20 to 40 cm deep for woody plant, within which the botanic roots grew vigorously. It has been confirmed that rhizosphere could assemble a great quantity of microbial communities living with the root exudates, and was abundant with nutrient, enzyme, water and oxygen^41^,^42^. The second-layer soil was approximately within the scope of the rhizosphere, therefore, it was an ideal site that nitrification, denitrification and respiration could proceed at high rates.

Nevertheless, although nitrification, denitrification and respiration rates in the third-layer soil were not the highest, these three microbial activities were most likely to happen in this layer. Most of the denitrification happened in the third-layer soil, as it could provide the most favourable anaerobic conditions. However, the rates of nitrification, denitrification and respiration might be constrained by the content of nutrient and organic matters in the third-layer soil, as studies have confirmed that nutrient and organic matters usually decreased with the increase of soil depth^43^,^44^.

During the field survey, we found that animal manure was applied to most of the vegetable gardens and the walnut forests. Moreover, the study area had a long history of manure application, especially to the vegetable garden. According to the research findings by many scholars, animal manure could have numerous gross effects on the population and structure of soil microbial communities^45^,^46^,^47^, as well as the physical properties of soils^48^. Therefore, high nitrification, denitrification and respiration rates in the vegetable garden and the walnut forests were likely to be attributed to the manure addition, which regulated microbial activities indirectly through optimizing the microbial communities and soil structures. Microbial nitrification, denitrification and respiration under any other vegetation covers (i.e., the corn field, apple orchard, chestnut forest and poplar forest) were less impressive but still varied considerably among each other. Except for the inhomogeneity and anisotropy of the leached cinnamon soil, plant species might also be the important reason for this phenomenon. Since plant species have been proven to be associated with soil properties and microbial compositions^49^. Additionally, fertilizer levels were various among the plant species, a large amount of field experiments have shown that the structure and the activity of microbial community had distinct features under different fertilization strategies^44^,^50^. Furthermore, from the overall point of view, nitrification, denitrification and respiration rates in the natural forest soil (i.e., poplar forest) were inferior to those in agricultural soils (i.e., vegetable garden, apple orchard, corn field, chestnut forest and walnut forest). This might be the implication that agriculture activities could enhance the activity of microorganism in the leached cinnamon soil, however, whether the enhanced the activity of microorganism would pose positive impacts on the environment needs to be further studied.

Soil physical and chemical characters such as pH, soil water content, soil organic matter content, C/N ratio, 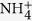 and 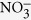 contents influence the presence of soil microbial community structure^51^, which could affect the rates of nitrification, denitrification and respiration. However, from our available data, no statistical significant dependence of nitrification, denitrification or respiration rate was observed on pH values, soil water contents, soil organic matter contents, C/N ratios, 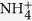 or 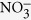 contents (see Supplementary Fig. S1). Possible explanations would be that the values of pH, soil water contents, C/N ratios and 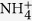 contents varied in a relative narrow range among all measurements (see Supplementary Table S1), which was insufficient to vindicate the variations of nitrification, denitrification and respiration rates. Moreover, soil organic matter content controls large-scale variations of nitrification, denitrification and respiration rates rather than site-specific variations.

The N_x_O_y_ represented a mixture of nitrogenous substances including N_2_O, NO and N_2_, yet N_2_O was the main gas will cause greenhouse effect. Many studies have revealed that nitrification and denitrification are the main processes for N_x_O_y_ production^21^,^22^,^23^,^24^. However, the more thoroughly the nitrification/denitrification process went on, the less proportion the N_2_O will be generated. In order to address the role of nitrification and denitrification in N_x_O_y_ emission, we conducted a regression analysis between denitrification rate and the sum of nitrification and denitrification rates. The regression function showed significant liner relationship (P < 0.0001, R^2^ = 0.908) between denitrification rate and the sum of nitrification and denitrification rates (see Supplementary Fig. S2). Denitrification rate averaged about two times of nitrification rate, thus denitrification rather than nitrification played a key role in producing N_x_O_y_ emission.

Moreover, in this paper, we could conclude that N_2_O was not the primary form of the N_x_O_y_, thus the soil and air temperatures in the sealed chamber did not increased correspondingly with the increasing of N_x_O_y_ emission. The increasing of air temperature was greater than that of the soil temperature in the sealed chamber. Accordingly, it indicated that most of the leached cinnamon soil samples had favourable porosity condition, therefore, a majority of CO_2_ and N_x_O_y_ was released, with the small proportions remained in the soil samples. For practical purposes, a number of possible measures could be adopted to reduce the emissions of CO_2_ and N_x_O_y_ from the leached cinnamon soil, such as: (a) researchers have proven that increase in soil pH could accelerate the removal of 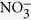 and immobile 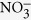 into organic N, thus reducing the emission of N_2_O^52^. According to our laboratory measurement, the leached cinnamon soil samples in the studied area are mildly acidic, with an average pH value of about 6.82. Therefore, proper amount of alkaline medium such as lime could be mixed into the soil to increase soil pH; (b) as denitrification is the principal way to produce N_2_O, meanwhile aerobic condition is an important factor limiting denitrification, therefore, plowing and scarifying the soil regularly could enhance land ventilation, thus inhibiting the proceeding of denitrification, as well as reducing the emission of N_2_O; and (c) recently, the use of biochar has been identified as an effective approach to reduce N_2_O emission, the decrease of soil N_2_O emission after biochar addition could achieve 10–90%^53^. Moreover, biochar is environmentally sound with little secondary pollution. Therefore, biochar could be an optimum choice for reducing N_2_O emission in the studied area.

Soil nitrogen availability has been defined as the amount of mineral nitrogen (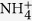 and 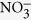) in soil which could be directly taken up by plants^54^,^55^,^56^. Microbial nitrification, denitrification and respiration played important roles in regulating soil nitrogen availability^57^,^58^,^59^. Theoretically, nitrification transformed the less mobile 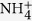 into a much more mobile form (i.e., 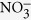), which was beneficial for promoting soil nitrogen availability. Conversely, denitrification consumes the 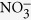 and produces nitrogenous gases (i.e., N_x_O_y_), by which soil nitrogen was lost in forms of gases, thus decreasing the soil nitrogen availability. Microbial respiration decomposes organic matter into CO_2_, the rate of CO_2_ production is a direct measure of microbial activity. Some studies have obtained a significant positive relationship between nitrogen mineralization rate and microbial respiration rate^60^,^61^, for the reason that mineralization processes involved in the N cycle are biotic, which would have higher rates with a higher microbial activity^62^. Therefore, nitrification, denitrification and respiration are three interrelated processes that affect the soil nitrogen availability.

**Figure 4:**
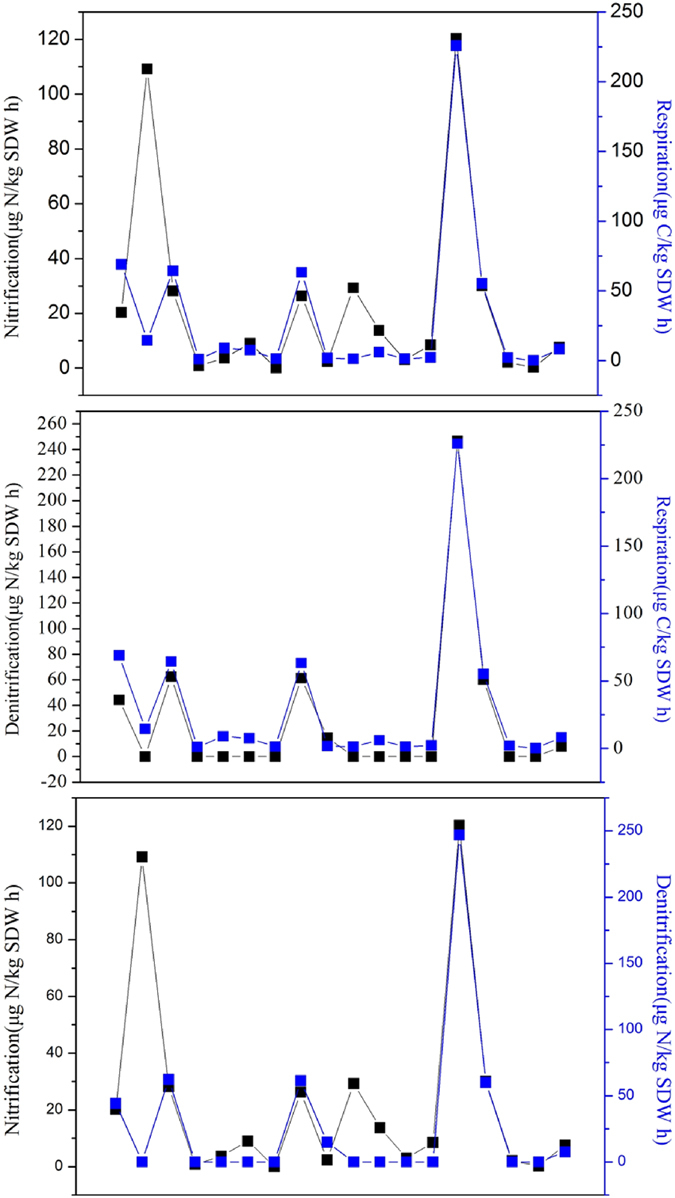
The relationship between nitrification, denitrification and respiration rates in the leached cinnamon soil samples.

We studied the relationships between the rates of nitrification, denitrification and respiration. Apparently, both nitrification and denitrification rates corresponded well with respiration rate (Fig. 4). However, denitrification rate tended to be more consistency with respiration rate than that of nitrification rate, for the reason that denitrification and respiration are carbon consuming processes^23^,^63^,^64^,^65^. As can be seen from Fig. 4, there existed high nitrification rate with low respiration rate. It might be because that: (1) both nitrification and respiration were oxygen-consumed processes, meanwhile nitrification was superior to respiration on oxygen competition; and (2) respiration process required organic matters, the lacking of organic matters in certain soil samples could also hinder the proceeding of respiration.

Results also showed that in the leached cinnamon soil samples, nitrification and denitrification were likely to be correlated and at the same time being affected by respiration. In most cases, nitrification occurred but denitrification seldom happened (Fig. 4). The favourable oxygen content could possibly be one of the reasons. However, with nitrification progressed, soil oxygen was depleted and 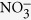 was accumulated, which were conductive to the occur of denitrification theoretically. But in reality respiration usually existed with nitrification, it might be that respiration consumed certain amount of soil organic matter, the surplus amount of soil organic matter was inadequate to initiate denitrification. Furthermore, nitrification and denitrification were co-existed under certain circumstances, with higher denitrification rate than nitrification rate (Fig. 4). It was not difficult to find that respiration also happened on this occasion. As nitrification and respiration progressed, soil oxygen was consumed and CO_2_ was generated, which provided profitable anaerobic condition for denitrification. Besides, the soil might contain sufficient 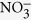 and organic matter, which could maintain the denitrification to proceed at high rates.

**Figure 5:**
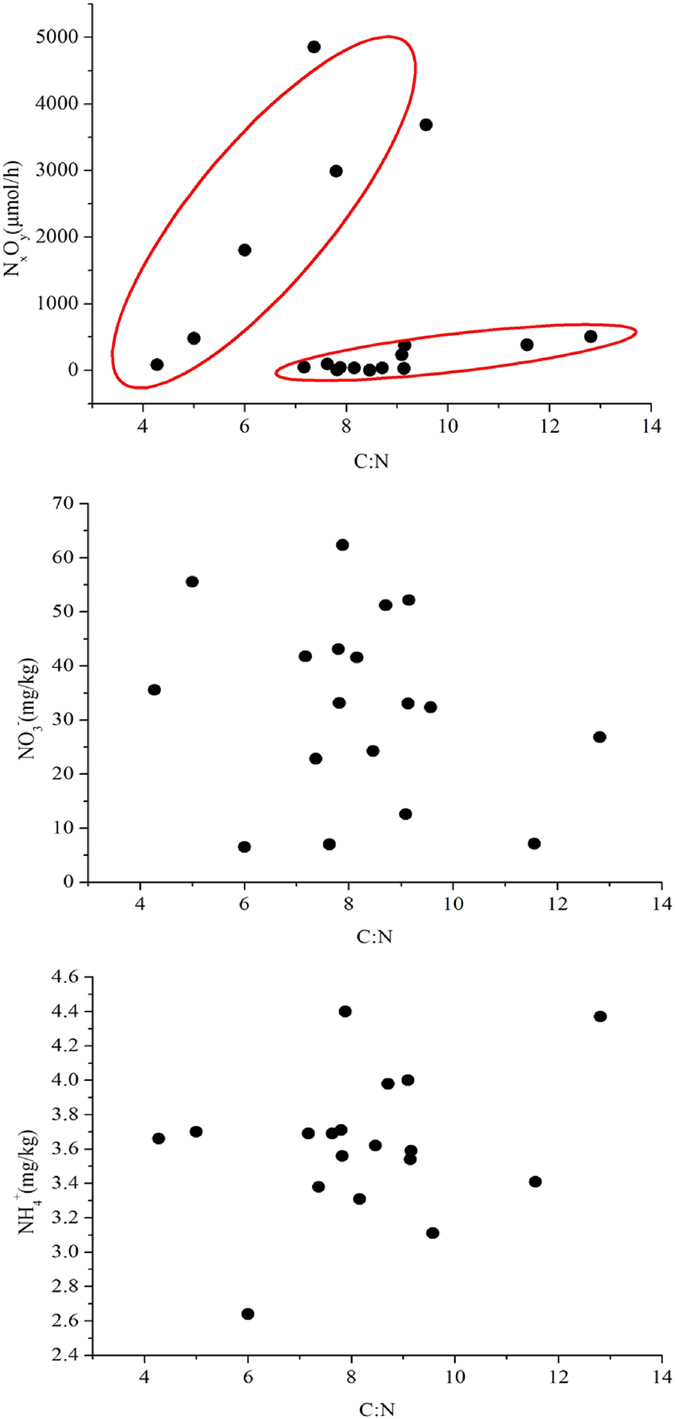
Variation of N_x_O_y_ emission rate, 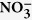 and 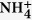 content with the carbon to nitrogen ratios in the leached cinnamon soil samples.

The C:N ratio has been regarded as an indicator of the qualitative changes of soil organic matter^66^, and has been found to have certain effects on soil N_2_O emission^67^. Therefore, it was a good reflection of the mutual interactions between soil carbon and nitrogen transformation processes. In the leached cinnamon soil samples, the total nitrogen content had a liner relationship with the total carbon content (see Supplementary Fig. S3), which indicated that the soil carbon and nitrogen transformation processes were linked tightly with each other. We also studied the influence of C:N ratio on the variation of N_x_O_y_ emission rate, 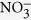 and 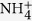 contents (Fig. 5), so as to analyze the impacts of carbon and nitrogen transformation processes on soil nitrogen availability. In the leached cinnamon soil samples, the value of C:N ratio varied from 4.27 to 12.81, with most of the values fluctuated around 7.16 to 9.57.

The emission rate of N_x_O_y_ increased with the C:N ratio, yet in a complex approach. A correlation was observed between the emission rate of N_x_O_y_ and the C:N ratio ranged from 4.00 to 8.00. However, the higher C:N ratio ranged from 7.00 to 13.00 merely corresponded to a slight variation of the emission rate of N_x_O_y_ (Fig. 5). It has been widely accepted that soil microorganism has a C:N ratio close to 8:1. In the leached cinnamon soil samples, observed results showed that the C:N ratio greater than 8:1 would result in a provisional nitrogen deficit in the soil. For the reason that soil microorganism might have to find additional nitrogen available in the soil to go with the excess carbon. On this occasion, soil nitrogen was privileged to participate in the immobilization processes, which contributed little to the emission of N_x_O_y_. Conversely, the C:N ratio less than 8:1 was likely to bring about a temporary nitrogen surplus in the soil. Because lower C:N ratios (≤8:1) represented a lesser proportion of carbon to nitrogen, thus soil microorganism would consume the organic matters in the soil and leave the excess nitrogen in the soil. Under this circumstances, soil nitrogen was increased through the mineralization processes. The surplus nitrogen in the soil would be available for plant assimilation, or for soil microorganisms to use to decompose other organic matters, as well as taking part in some microbial processes such as nitrification and denitrification, which were the dominant contributors to the emission of N_x_O_y_.

Nevertheless, no correlation was found between C:N ratio and 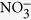 content or between C:N ratio and 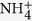 content (Fig. 5). Possible reasons were that: (1) soil 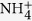 and 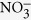 could be originated from both exogenous sources (i.e., anthropogenic fertilizer addition) and endogenous sources (i.e., soil organic matter mineralization); and (2) soil 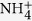 and 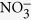 were indispensable reactants of microbial nitrification and denitrification, respectively. In certain cases, microbial nitrification and denitrification were co-existed and had mutual influences on each other. Moreover, 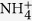 could be oxidized into 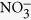. In aggregate, both of the reasons enhanced the complexities and uncertainties in describing the relationship between C:N ratio with 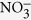 and 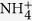 content, respectively. Hence, there did not exist correlations between C:N ratio with neither 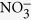 nor 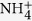 content.

**Figure 6:**
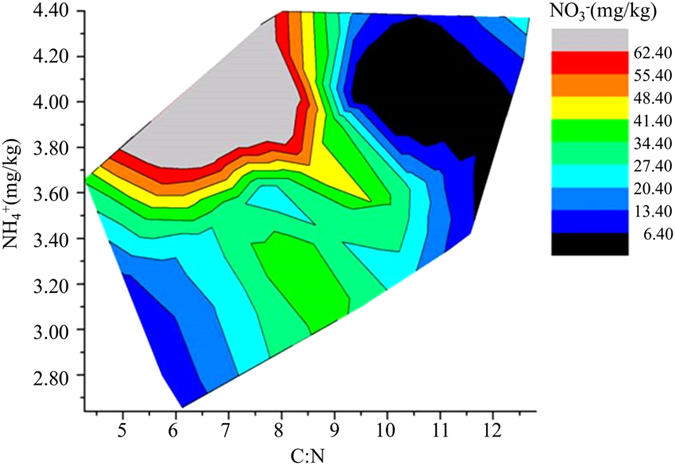
Contour map of the 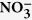 content as influenced by both 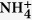 content and C:N ratio in the leached cinnamon soil samples.

A number of studies have consistently found that 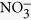 is the dominant form of nitrogen present in the soil to provide the plant-available nitrogen^68^,^69^. However, the content of 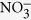 in the soil could be affected by many factors (i.e., soil properties, microbial activities, and human disturbances). Therefore, it would benefit a lot if there were accessible indicators that could imply the availability of soil nitrogen (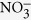 mostly). Tracing back to the above research and analysis, we tried the C:N ratio as an indicator of the 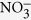 content, yet no correlation was observed between these two variables. Nevertheless, we analysed that 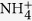 and 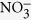 content were somehow connected. Therefore, we tried to study the variation of 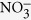 content with both C:N ratio and 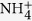 content (Fig. 6).

It could be seen that 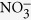 content was indeed affected by the C:N ratio and 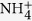 content together. Soil 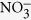 content was the most sufficient (>62.4 mg/kg) when the C:N ratio ranged from 5.30 to 8.40, meanwhile the 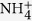 content was between 3.71 and 4.39 mg/kg. However, soil 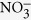 content was the poorest (<6.40 mg/kg) when the C:N ratio varied from 9.25 to 12.10, with the 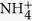 content changed between 3.41 and 4.35 mg/kg (Fig. 6). Possible reasons were that: (1) as already discussed, higher C:N ratio (more than 8:1) in the leached cinnamon soil samples might lead to the immobilization of soil nitrogen, while lower C:N ratio (less than 8:1) could facilitate the mineralization of soil organic matters thus resulting in the surplus of soil nitrogen; (2) the 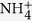 content in the leached cinnamon soil remained between 2.64 and 4.40 mg/kg, despite the ammonia loss by volatilization, microbial nitrification might be another approach to consume the 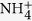. And we have analyzed that microbial nitrification was pervasively happened in the leached cinnamon soil samples at different rates (0.84 to 120.35 μg N/kg SDW h). Based on the statistical analysis, higher 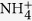 level (3.41 and 4.35 mg/kg) were more likely to maintain nitrification at high rates (i.e., 109.18 and 120.35 μg N/kg SDW h), thus contributing more 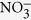 to the soil.

## Methods

### Site description

This research was conducted at the Tumenxigou watershed (E115°25′–117°35′, N40°19′–41°38′), which is located in Miyun county and is about 5 km away from the northeast of Beijing city. The entire watershed covers an area of approximately 3.49 km^2^, and has a warm temperate and continental monsoon climate. The annual mean temperature in this region is 10.8 °C, and the multi-year average precipitation is about 660 mm. The main landform includes low mountains and hills, with the altitude ranges from 242.0 to 781.4 m. Soil textures are comprised of leached cinnamon soil, loamy soil, gravel soil and sandy soil. Nevertheless, leached cinnamon soil is the predominant layer for plant growth, with a depth to about 50 to 60 cm, thus being selected as the study soil type in this research. Generally, leached cinnamon soil is weakly alkaline, with the pH value being around 7.30. However, agricultural practices could alter the value of soil pH to a certain extent. According to our laboratory measurement, the leached cinnamon soil samples in the studied area are mildly acidic, with an average pH value of about 6.82. The average content of soil organic matter and total nitrogen are 12.85 g/kg and 0.70 g/kg, respectively. Based on field surveys, the major vegetation cover types within this area could be devided into six categories, including the vegetable garden, corn field, apple orchard, chestnut forest, walnut forest and poplar forest, respectively.

### Experimental design

**Figure 7:**
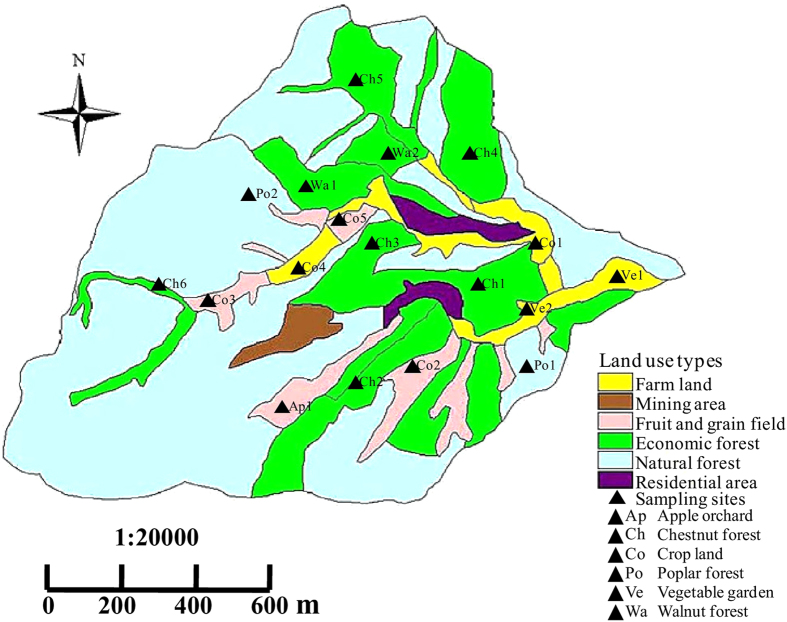
Sampling locations in the study watershed. This map was created by myself via the software called ArcGIS9.3, of which a 60-day free trial version could be download at the website as following: http://www.esri.com/software/arcgis/arcgis-for-desktop.

In the late July of 2015, 18 soil sampling points were identified with the systematic random sampling method based on the area of each vegetation cover. In detail, there were two sampling points in the vegetable garden, five sampling points in the corn field, one sampling point in the apple orchard, six sampling points in the chestnut forest, two sampling points in the walnut forest and two sampling points in the poplar forest (Fig. 7). On each sampling site, undisturbed soil samples were collected with the ring samplers (i.e., Φ_1_ = 56 mm, Φ_2_ = 60 mm, and h = 40.5 mm), which is made of stainless steel, cylinder-shaped, and open at both ends (one of the end is edged, which is drilled into soil when sampling). According to the buried depth of plant rhizosphere, we collected soil samples in depth of 0–10, 10–20 and 20–30 cm in the vegetable garden and the corn field, respectively. As for the apple orchard, walnut forest, chestnut forest and poplar forest, soil samples were collected in depth of 0–20, 20–40 and 40–60 cm, respectively. Moreover, three parallel samples were collected in each sampling sites for laboratory use afterwards. Soil samples were sealed in the ring samplers with plastic caps at both ends, and were kept in the refrigerator at 4 °C until analytical test. Then, Soil samples were incubated in a sealed chamber at 25 °C (close to the soil temperature at field). After 12 hours’ incubation, the nitrification, denitrification and respiration rates, as well as the CO_2_ and N_x_O_y_ emission rates of the leached cinnamon soil samples were determined. In our research, soil samples were taken back to laboratory for measurement with the BaPS (Barometric Process Separation) method. Since roots in the soil samples became inactive as being separated from live plants, autotrophic (i.e., roots) respiration became negligible compared to heterotrophic (i.e., microbes and soil fauna) respiration. Therefore, only microbial processes (i.e., nitrification, denitrification and respiration) were considered in this research.

### Measurement of the nitrification, denitrification and respiration rates

The BaPS (Barometric Process Separation) method is applied to determine the rates of nitrification, denitrification and respiration. Soil samples were incubated in a sealed chamber at 25 °C (close to the soil temperature at field) for at least 12 hours. The barometric changes in the sealed chamber were mainly induced by the processes of microbial nitrification, denitrification and respiration. The three microbial processes could be generalized as follows, respectively:


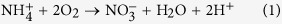



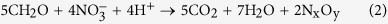



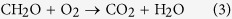


The CO_2_ sensor and the pressure sensor of the BaPS system monitored the dynamic equilibrium of CO_2_, N_x_O_y_ production, and O_2_ depletion every ten minutes, as well as the variation of the gross gas volume (Δn):


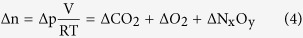


In equation 4, ΔCO_2_, ΔO_2_, Δp and T are important parameters that measured with the BaPS method directly, at the end of incubation, the BaPS system will automatically calculate the potential rates of nitrification, denitrification and respiration based on the dynamic changes of these parameters (i.e., ΔCO_2_, ΔO_2_, Δp and T), the detailed derivation process is referenced to Ingwersen *et al*.^36^. The detection limit of ΔCO_2_, ΔO_2_, Δp and T are 0–3 Vol%, 15–21 Vol%, 800–1200 hpa and −30–70 °C, respectively. The precision degree of ΔCO_2_, ΔO_2_, Δp and T are ±2%, ±1%, ±0.1% and 0.1 °C, respectively.

### Auxiliary measurements

We measured the C:N ratios of the leached cinnamon soil samples using the elemental analyzer (EA3000., Italy). Soil samples were air-dried and grinded before passing through a 100-mesh sieve. Three parallel 25 mg sifted soil samples were weighed and poured into tin capsules. The tin capsules containing soil samples were shaped into balls for the elemental analyzer to test. The N (%) and (C%) contents of the soil samples were determined using the elemental analyzer at high-temperature combustion (980 °C). The C:N ratios were calculated based on the values of N (%) and (C%) contents.

We measured the 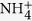 and 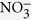 concentrations of the leached cinnamon soil samples using the flow analyzer (AA3., Germany). Three parallel 5.0 g fresh soil samples were weighted and extracted with 25 mL of freshly prepared 2 M KCL (soil : water ratio of 1:5), capped and shaken at 300 rpm for 1 h. Then, the suspensions of the soil solutions were kept stand still for 30 minutes, after which the supernatants were filtered through 0.45 μm nitrocellulose membrane filters. The filtered soil solutions were injected into 10 mL centrifuge tubes. Afterwards, 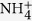 and 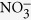 concentrations of the filtered soil solutions were determined by the flow analyzer. 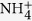 and 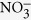 concentrations of the soil samples could be calculated by the following equation:


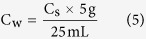


where C_W_ means the 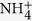 or 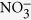 concentrations of the filtered soil solutions, mg/L; C_s_ means the 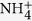 or 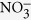 concentrations of the soil samples, mg/Kg.

## Additional Information

**How to cite this article:** Xu, W. *et al*. Microbial nitrification, denitrification and respiration in the leached cinnamon soil of the upper basin of Miyun Reservoir. *Sci. Rep.* **7**, 42032; doi: 10.1038/srep42032 (2017).

**Publisher's note:** Springer Nature remains neutral with regard to jurisdictional claims in published maps and institutional affiliations.

## Supplementary Material

Supplementary Information

## Figures and Tables

**Table 1:** Variations of the gas volume and soil/air temperatures after 12 h’s incubation in the BaPS system.

	Soil depth (cm)	Vegetation types	Δn (μmol/h)	ΔCO_2_ (μmol/h)	ΔO_2_ (μmol/h)	ΔN_x_O_y_ (μmol/h)	ΔT_soil_ (°C)	ΔT_air_ (°C)
The first layer soil	0–10	Vegetable garden	252.20	547.80	−669.30	373.70	0.17	0.08
		Corn field	−45.30	3.70	−15.00	−34.00	0.16	0.26
	0–20	Apple orchard	0.00	13.00	−13.00	0.00	−0.13	−0.72
		Chestnut forest	−49.80	1.60	−12.00	−39.30	0.03	0.03
		Walnut forest	−45.47	2.43	−12.57	−35.33	0.17	0.18
		Poplar forest	−58.40	2.80	−15.20	−45.90	0.16	0.35
The second layer soil	10–20	Vegetable garden	−1305.20	89.40	−319.50	−3685.50	0.38	0.56
		Corn field	−650.50	98.20	−242.50	−506.20	0.31	0.50
	20–40	Apple orchard	440.30	148.90	−90.60	382.10	0.26	0.80
		Chestnut forest	−1081.83	126.90	−383.55	−2988.85	0.25	0.63
		Walnut forest	4264.30	116.70	−703.05	4850.65	0.16	0.76
		Poplar forest	−18.60	2.40	−15.00	−6.00	0.20	0.38
The third layer soil	20–30	Vegetable garden	263.70	187.50	−155.60	231.80	0.45	1.00
		Corn field	−533.20	124.60	−178.60	−479.20	0.40	0.39
	40–60	Apple orchard	222.90	215.50	−77.20	84.60	0.33	0.14
		Chestnut forest	−33.90	1.00	−8.10	−26.90	0.12	0.25
		Walnut forest	1466.50	23.70	−362.20	1805.00	0.39	1.27
		Poplar forest	89.90	8.10	−11.30	93.10	0.18	0.64
