# Supplementary material for: Microbial nitrification, denitrification and respiration in the leached cinnamon soil of the upper basin of Miyun Reservoir
Source: Sci Rep. 2017 Feb 6;7:42032. doi: 10.1038/srep42032 (PMC5292740; doi:10.1038/srep42032)
Supplement: Supplementary Information [file srep42032-s1.pdf]

Microbial nitrification, denitrification and respiration in the leached cinnamon soil of the upper basin of Miyun Reservoir

Wen Xu[a], Yan-Peng Cai[a,b,c*], Zhi-Feng Yang[a,b], Xin-An Yin[a,b], Qian Tan [c]

a. State Key Laboratory of Water Environment Simulation, School of Environment, Beijing Normal University, Beijing, China, 100875; b. Beijing Engineering Research Center for Watershed Environmental Restoration & Integrated Ecological Regulation, School of Environment, Beijing Normal University, Beijing, China, 100875; c. Institute for Energy, Environment, and Sustainable Communities, University of Regina, Research Drive, Regina, Saskatchewan S4S 7H9, Canada.

*Corresponding author: State Key Laboratory of Water Environment Simulation, School of Environment, Beijing Normal University, Beijing, China, 100875; Email address: yanpeng.cai@ bnu.edu.cn, Tel.: +86 10 58800830, Fax.: +86 10 58802795

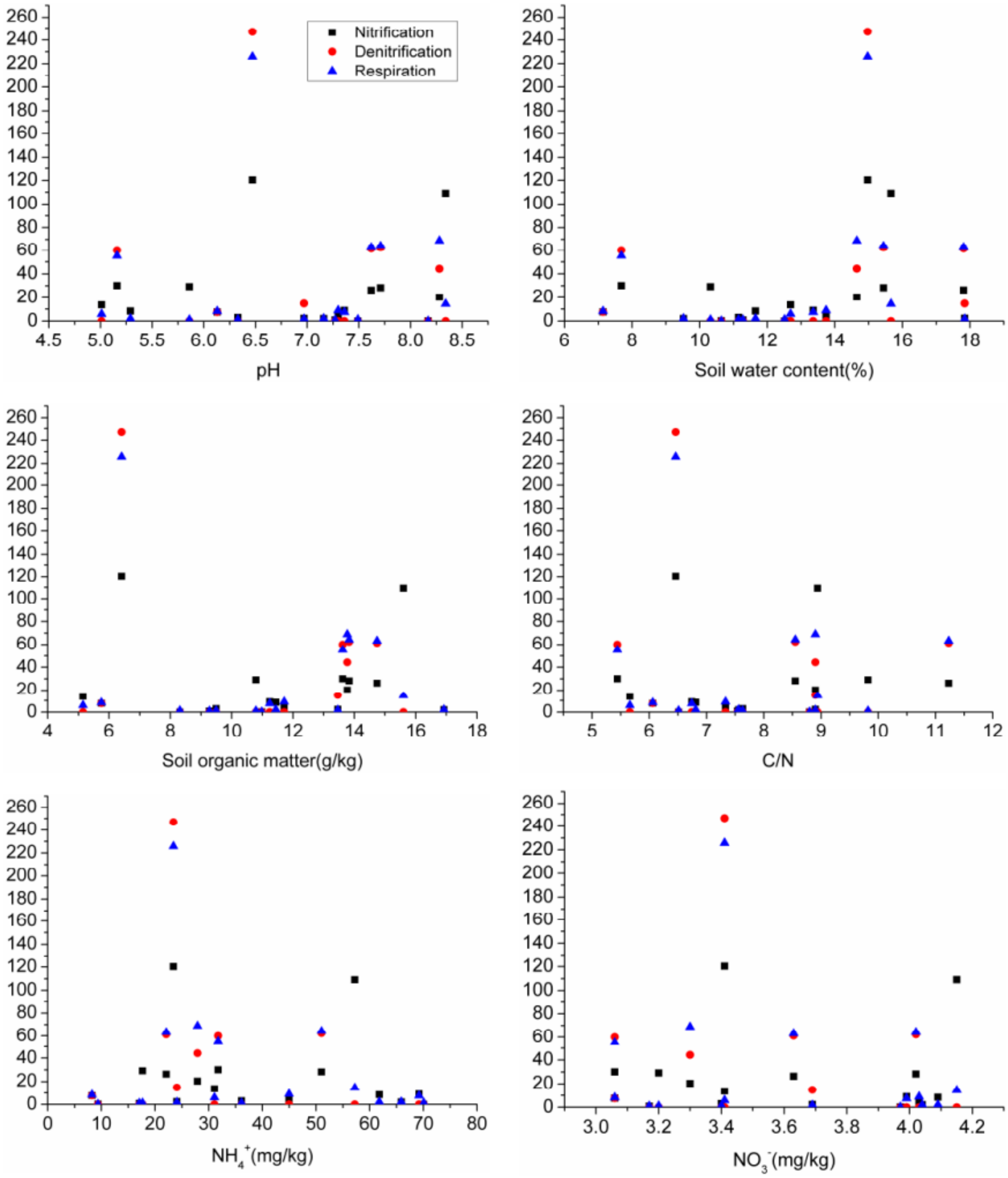

Supplementary Figure S1. Relationships between some physical/chemical characters and the nitrification, denitrification and respiration rates

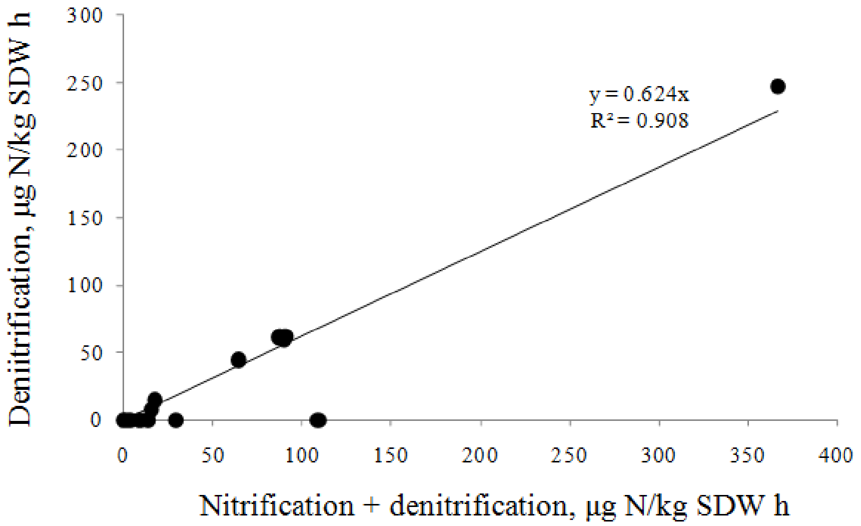

Supplementary Figure S2. Regression analysis between denitrfication rate and the sum of nitrification and denitrification rates

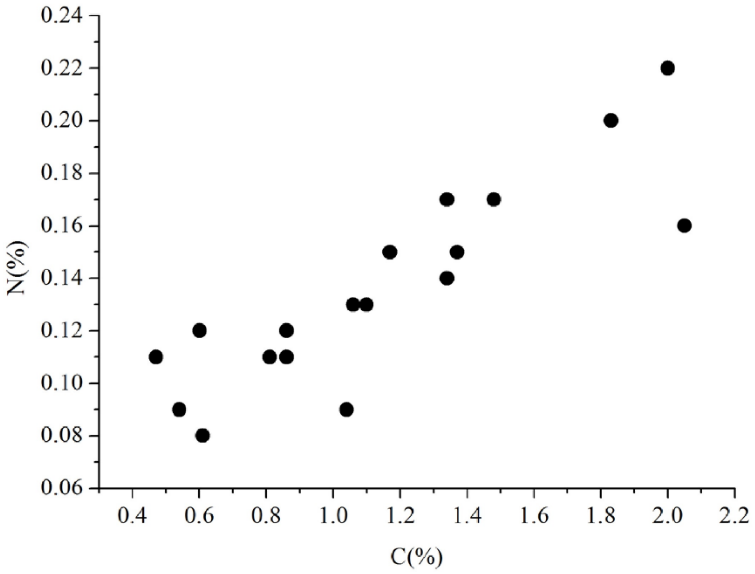

Supplementary Figure S3. Relationship between the content of total nitrogen and total carbon in the leached cinnamon soil samples

Supplementary Table S1. The pH values, soil water contents, soil organic matter contents, C/N ratios, NH_4^+ and NO_3^- contents of the soil samples

Sample number	Sampling field	pH	Soil water content (%)	Soil organic matter (g/kg)	C/N ratio	NH_4^+ (mg/kg)	NO_3^- (mg/kg)
1	Vegetable garden	8.28	14.65	13.77	8.90	3.30	27.90
2	Vegetable garden	8.34	15.66	15.60	8.94	4.15	57.21
3	Corn field	7.71	15.44	13.83	8.55	4.02	51.02
4	Corn field	7.27	11.28	9.27	7.54	3.17	17.06
5	Corn field	7.30	13.74	11.71	7.33	4.03	44.97
6	Corn field	7.36	13.36	11.24	6.74	3.99	69.19
7	Corn field	7.49	12.51	8.31	6.51	4.04	70.07
8	Apple orchard	7.62	17.81	14.74	11.23	3.63	22.08
9	Chestnut forest	6.97	17.84	13.46	8.90	3.69	24.05
10	Chestnut forest	5.86	10.32	10.79	9.82	3.20	17.66
11	Chestnut forest	5.01	12.69	5.15	5.66	3.41	31.06
12	Chestnut forest	6.33	11.15	9.49	7.63	3.40	36.12
13	Chestnut forest	5.29	11.65	11.44	6.81	4.09	61.78
14	Chestnut forest	6.47	14.97	6.41	6.46	3.41	23.42
15	Walnut forest	5.16	7.68	13.62	5.44	3.06	31.76
16	Walnut forest	7.16	9.52	16.92	7.56	4.04	65.89
17	Poplar forest	8.17	10.64	10.98	8.80	3.97	9.37
18	Poplar forest	6.13	7.14	5.75	6.06	3.06	8.24
